# Supplementary material for: Modeling glioblastoma heterogeneity as a dynamic network of cell states
Source: Mol Syst Biol. 2021 Sep 16;17(9):e10105. doi: 10.15252/msb.202010105 (PMC8444284; doi:10.15252/msb.202010105)
Supplement: Supplementary file 6 — Source Data for Figure 5 [file MSB-17-e10105-s004.zip › Figure5A_sourcedata/GSEA_3071/hallmarks_stateVI.GseaPreranked.1623742557031/HALLMARK_UV_RESPONSE_DN.html]

Details for gene set HALLMARK\_UV\_RESPONSE\_DN[GSEA]

|  || Dataset | state63071 |
| Phenotype | NoPhenotypeAvailable |
| Upregulated in class | na\_pos |
| GeneSet | HALLMARK\_UV\_RESPONSE\_DN |
| Enrichment Score (ES) | 0.3471886 |
| Normalized Enrichment Score (NES) | 2.2120128 |
| Nominal p-value | 0.0 |
| FDR q-value | 0.0011636365 |
| FWER p-Value | 0.002 |
Table: GSEA Results Summary

  

Fig 1: Enrichment plot: HALLMARK\_UV\_RESPONSE\_DN      
 Profile of the Running ES Score & Positions of GeneSet Members on the Rank Ordered List

  

| PROBE | GENE SYMBOL | GENE\_TITLE | RANK IN GENE LIST | RANK METRIC SCORE | RUNNING ES | CORE ENRICHMENT || 1 | SERPINE1 |  |  | 0 | 1.673 | 0.0421 | Yes |
| 2 | CITED2 |  |  | 5 | 1.232 | 0.0721 | Yes |
| 3 | MT1E |  |  | 10 | 1.128 | 0.0994 | Yes |
| 4 | CAV1 |  |  | 13 | 1.087 | 0.1262 | Yes |
| 5 | F3 |  |  | 26 | 0.988 | 0.1477 | Yes |
| 6 | RND3 |  |  | 49 | 0.866 | 0.1634 | Yes |
| 7 | KCNMA1 |  |  | 55 | 0.861 | 0.1837 | Yes |
| 8 | BDNF |  |  | 65 | 0.828 | 0.2021 | Yes |
| 9 | MYC |  |  | 69 | 0.815 | 0.2217 | Yes |
| 10 | DAB2 |  |  | 78 | 0.788 | 0.2394 | Yes |
| 11 | ANXA2 |  |  | 79 | 0.783 | 0.2591 | Yes |
| 12 | LTBP1 |  |  | 84 | 0.779 | 0.2776 | Yes |
| 13 | DLC1 |  |  | 129 | 0.696 | 0.2828 | Yes |
| 14 | DUSP1 |  |  | 136 | 0.685 | 0.2984 | Yes |
| 15 | IGFBP5 |  |  | 145 | 0.671 | 0.3131 | Yes |
| 16 | MET |  |  | 174 | 0.626 | 0.3210 | Yes |
| 17 | MGLL |  |  | 190 | 0.609 | 0.3322 | Yes |
| 18 | ADORA2B |  |  | 192 | 0.608 | 0.3472 | Yes |
| 19 | SYNJ2 |  |  | 270 | 0.555 | 0.3396 | No |
| 20 | SNAI2 |  |  | 310 | 0.536 | 0.3422 | No |
| 21 | NR3C1 |  |  | 395 | 0.492 | 0.3311 | No |
| 22 | FHL2 |  |  | 397 | 0.491 | 0.3432 | No |
| 23 | LPAR1 |  |  | 575 | 0.432 | 0.3046 | No |
| 24 | IRS1 |  |  | 614 | 0.421 | 0.3046 | No |
| 25 | PIK3CD |  |  | 615 | 0.421 | 0.3152 | No |
| 26 | FZD2 |  |  | 733 | 0.389 | 0.2923 | No |
| 27 | EFEMP1 |  |  | 763 | 0.384 | 0.2938 | No |
| 28 | NEK7 |  |  | 920 | 0.357 | 0.2592 | No |
| 29 | PDLIM5 |  |  | 1062 | 0.335 | 0.2282 | No |
| 30 | ATP2B4 |  |  | 1092 | 0.331 | 0.2285 | No |
| 31 | TFPI |  |  | 1205 | 0.315 | 0.2051 | No |
| 32 | RGS4 |  |  | 1209 | 0.314 | 0.2122 | No |
| 33 | MRPS31 |  |  | 1255 | 0.309 | 0.2074 | No |
| 34 | TGFBR3 |  |  | 1846 | -0.256 | 0.0488 | No |
| 35 | COL11A1 |  |  | 1919 | -0.267 | 0.0354 | No |
| 36 | HAS2 |  |  | 2025 | -0.282 | 0.0131 | No |
| 37 | DLG1 |  |  | 2065 | -0.290 | 0.0095 | No |
| 38 | FYN |  |  | 2118 | -0.301 | 0.0026 | No |
| 39 | COL5A2 |  |  | 2160 | -0.307 | -0.0012 | No |
| 40 | APBB2 |  |  | 2231 | -0.319 | -0.0127 | No |
| 41 | CDON |  |  | 2233 | -0.320 | -0.0049 | No |
| 42 | SIPA1L1 |  |  | 2427 | -0.360 | -0.0498 | No |
| 43 | LAMC1 |  |  | 2519 | -0.382 | -0.0656 | No |
| 44 | BMPR1A |  |  | 2555 | -0.389 | -0.0656 | No |
| 45 | IGF1R |  |  | 2618 | -0.404 | -0.0728 | No |
| 46 | MAGI2 |  |  | 2624 | -0.406 | -0.0640 | No |
| 47 | ARHGEF9 |  |  | 2681 | -0.419 | -0.0691 | No |
| 48 | PRKCA |  |  | 2847 | -0.474 | -0.1032 | No |
| 49 | ATXN1 |  |  | 2860 | -0.481 | -0.0945 | No |
| 50 | AKT3 |  |  | 2876 | -0.486 | -0.0864 | No |
| 51 | PIK3R3 |  |  | 2903 | -0.495 | -0.0812 | No |
| 52 | AMPH |  |  | 2981 | -0.529 | -0.0894 | No |
| 53 | ATRX |  |  | 3036 | -0.557 | -0.0905 | No |
| 54 | SDC2 |  |  | 3080 | -0.578 | -0.0880 | No |
| 55 | SRI |  |  | 3179 | -0.643 | -0.0992 | No |
| 56 | INPP4B |  |  | 3223 | -0.692 | -0.0938 | No |
| 57 | PLPP3 |  |  | 3394 | -0.880 | -0.1191 | No |
| 58 | CDKN1B |  |  | 3426 | -0.951 | -0.1038 | No |
| 59 | COL3A1 |  |  | 3430 | -0.960 | -0.0805 | No |
| 60 | NFIB |  |  | 3508 | -1.145 | -0.0732 | No |
| 61 | MMP16 |  |  | 3511 | -1.150 | -0.0448 | No |
| 62 | CELF2 |  |  | 3550 | -1.361 | -0.0211 | No |
| 63 | COL1A2 |  |  | 3602 | -1.803 | 0.0101 | No |
Table: GSEA details [plain text format]

  

Fig 2: HALLMARK\_UV\_RESPONSE\_DN: Random ES distribution      
 Gene set null distribution of ES for **HALLMARK\_UV\_RESPONSE\_DN**
